# Value-based cost-cognizant test case prioritization for regression testing

**DOI:** 10.1371/journal.pone.0264972

**Published:** 2022-05-17

**Authors:** Farrukh Shahzad Ahmed, Awais Majeed, Tamim Ahmed Khan, Shahid Nazir Bhatti

**Affiliations:** 1 Department of Software Engineering, Bahria University, Islamabad, Pakistan; 2 Department of Software Engineering, College of Computer Science & Engineering (CCSE), University of Jeddah, KSA, Jeddah, Saudi Arabia; Al Mansour University College-Baghdad-Iraq, IRAQ

## Abstract

Software Test Case Prioritization (TCP) is an effective approach for regression testing to tackle time and budget constraints. The major benefit of TCP is to save time through the prioritization of important test cases first. Existing TCP techniques can be categorized as value-neutral and value-based approaches. In a value-based fashion, the cost of test cases and severity of faults are considered whereas, in a value-neutral fashion these are not considered. The value-neutral fashion is dominant over value-based fashion, and it assumes that all test cases have equal cost and all software faults have equal severity. But this assumption rarely holds in practice. Therefore, value-neutral TCP techniques are prone to produce unsatisfactory results. To overcome this research gap, a paradigm shift is required from value-neutral to value-based TCP techniques. Currently, very limited work is done in a value-based fashion and to the best of the authors’ knowledge, no comprehensive review of value-based cost-cognizant TCP techniques is available in the literature. To address this problem, a systematic literature review (SLR) of value-based cost-cognizant TCP techniques is presented in this paper. The core objective of this study is to combine the overall knowledge related to value-based cost-cognizant TCP techniques and to highlight some open research problems of this domain. Initially, 165 papers were reviewed from the prominent research repositories. Among these 165 papers, 21 papers were selected by using defined inclusion/exclusion criteria and quality assessment procedures. The established questions are answered through a thorough analysis of the selected papers by comparing their research contributions in terms of the algorithm used, the performance evaluation metric, and the results validation method used. Total 12 papers used an algorithm for their technique but 9 papers didn’t use any algorithm. Particle Swarm Optimization (PSO) Algorithm is dominantly used. For results validation, 4 methods are used including, Empirical study, Experiment, Case study, and Industrial case study. The experiment method is dominantly used. Total 6 performance evaluation metrics are used and the APFDc metric is dominantly used. This SLR yields that value-orientation and cost cognition are vital in the TCP process to achieve its intended goals and there is great research potential in this research domain.

## 1. Introduction

Making great decisions in Software Engineering (SE) require a thorough understanding of the business consequences of those decisions [1]. SE research is primarily based on value-neutral settings in which all software artifacts have equal importance and there are many limitations of this fashion [2]. Value-based SE (VBSE) has catered to these limitations by considering value in software development principles and practices [2]. According to Barry Boehm, the definition of VBSE is “the explicit concern with value concerns in the application of science and mathematics by which the properties of computer software are made useful to people” [3]. The early research in the currently popular approach of agile software development was focused on extreme programming, pair programming, and lean software development. Now, there is a transition in this trend and the focus is on the value of developed features and continuous value delivery [4]. Current trends are focused on continuous value delivery and close coordination between business units and technical teams [4]. According to [5] there is a value-based view of software product quality. Software customers usually take the value-based view, and they are concerned about the value added by software products to their organization. They perform a cost-benefit analysis. This requires a good definition of customer expectations regarding software quality in terms of some value. To meet customers’ software quality expectations in terms of value, software testing becomes more critical. Software testing is an expensive and critical phase of the software development life cycle and often consumes 40–50% of the overall budget of a software project [6]. Software testing has become a critical part of software development, due to its complexity, size, and support to real-time businesses [7]. Just like other software development phases, software testing research is also primarily based on a value-neutral fashion in which all the code statements, requirements, use cases, conditions, methods, and scenarios are treated as equally important [8]. In value-neutral software testing, resources are allocated to the activities that are inefficient in the context of Return on Investment (ROI). Software testing costs around $300 billion a year worldwide [3]. Value-neutral testing is not directly linked to the business objectives of the product and is considered agnostic to value considerations [9]. To address these challenges, value-based testing was introduced.

Value-based testing involves testing software systems that can better align testing resources to meet the value objectives of the project [8]. The major thing in value-based testing is to integrate internal testing objectives with the client’s business objectives and expectations [8]. The focus is on value delivery instead of verifying code against a set of requirements. According to [2], the value-neutral testing generated a higher ROI of 1.22 with 100% test execution, and the value-based testing produced a higher ROI of 1.74 with the execution of about 40% of the most valuable test cases. This indicates that testing resources should be utilized in such a way that they can add value to the client’s business success. The tests should be aligned with the written requirements as well as with the client’s expectations. Therefore, testing activities should adopt a business value-based perspective. The value-based verification and validation are also included in the agenda of VBSE [10].

**Fig 1:**
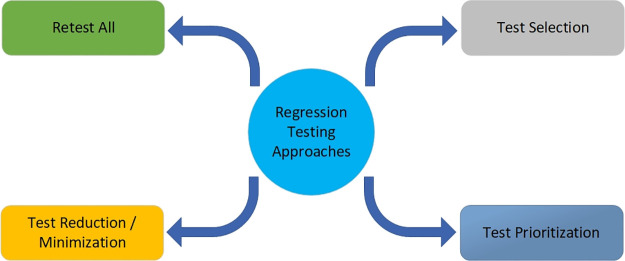
Regression testing approaches.

According to Boehm software cost is estimated at $1 trillion per year and testing activities cost half of this total cost. He said that 60% of the testing cost can be diminished and there is a cost-saving potential of $300 billion worldwide in a year through this value-based testing investment [3]. Regression testing is among the most expensive testing activities and is a big challenge in rapidly growing and changing systems. It consumes a large amount of time as well as effort and mostly accounts for around half of the software maintenance costs [11]. Regression testing is normally associated with system testing after any code change, it can be carried out at the system level, integration level, or unit level [11]. Complete test coverage is normally not possible during regression testing due to limited time and resources. Then the question arises: how much regression testing is enough, which is always a challenge for the testing teams. There are four types of techniques used in regression testing for test case coverage including TCP, test case selection, test case reduction, and retest all [12, 13]. Fig 1 illustrates the different types of regression testing approaches.

The test selection approach is widely applied in the industry, but it is not risk-free because it is based on selection. Similarly, the test case reduction cannot guarantee that only unrelated test cases are eliminated from the test case pool. On the other hand, TCP does not reduce or remove test cases from the test suite. That is why it is more secure, reliable, and popular in practice and a lot of research work is being done in this field.

**Test case prioritization** is one of the ways for optimized regression testing [14]. Rothermel *et al*. defined the TCP problem as follows [15]. *Suppose T is a test suite*, *PT is a set of permutations of T*, *and f is a function from PT to real numbers*, *f*: *PT→R*.

*Prioritization Goal*: *To find a T*^*I*^ *∈ PT that maximizes f*.

The factors that are considered in TCP techniques include the size of the test case set, cost, time, effort, efficiency, number of defects detected, and repetitiveness [16]. Different TCP techniques have been primarily proposed to increase the Average Percentage of Fault Detection (APFD), by prioritizing the test cases to save time and cost. There are two different fashions in which TCP techniques have been proposed. These fashions include value-based and value-neutral. In a value-based fashion, the cost of test cases and the severity of bugs are considered while prioritizing test cases for regression testing. Whereas the value-neutral fashion assumes that all faults have equal severity, and all test cases have equal cost. But in practice this assumption rarely holds. Value-neutral fashion is dominant over value-based fashion.

The value-neutral TCP techniques consider that all faults have the same severity, and all test cases have the same cost. Similarly, the metrics used for performance evaluation of TCP techniques like Average Percentage of Statement Coverage (APSC), the Average Percentage of Fault Detection (APFD), the Total Percentage of Fault Detection (TPFS), the Average Percentage of Branch Coverage (APBC), the Average Percentage of Function Coverage (APFC), the Average Percentage of Condition Coverage (APCC), and the Average Percentage of X elements Coverage (APXC) are also proposed in a value-neutral fashion. All these metrics assume that all faults have the same severity, all requirements have the same worth, and all code statements have the same value but practically this is very rare. Different bugs have different severity, and different requirements have different values. Similarly, the value of different functions, statements, conditions, branches, and methods may differ from other functions, statements, conditions, branches, and methods, respectively. Most of the existing TCP techniques are coverage-based and are effective at unit level testing, but are time-consuming and consider that all bugs are equally severe and all test cases have equal cost [5]. This assumption is not possible in practice [17]. A summary of the existing value-neutral TCP techniques along with their core objectives is presented in Table 1.

**Table 1:** Summary of TCP techniques.

Year	Author	TCP objectives	Category	Ref.
2008	Heiko *et al*.	Test case generation from activity diagram and do risk-based prioritization.	Risk-based	[18]
2010	Askarunisa *et al*.	Prioritizes tests by using sequences of XML messages. Effective for fault detection for composite web services.	History-based	[19]
2011	Wang *et al*.	Risk-based regression testing that detects most potential bugs with the minimum test cases. Saves computational resources and time using the Genetic Algorithm (GA).	Risk-based	[20]
2012	H. Mei *et al*.	A static TCP approach is proposed for the prioritization of the unit test case.	Coverage-based	[21]
2013	Ning *et al*.	A puzzle-based technique that improves branch coverage by decomposing object mutation and constraints solving problems into small puzzles.	Coverage-based	[22]
2013	Ti *et al*.	History-based technique for better fault detection through version awareness.	History-based	[23]
2014	Rongxin *et al*.	Crash locating-based TCP method to uncover crash scenarios in the application through crash reports.	History-based	[24]
2015	Geetanjali *et al*.	Clustering-based novel TCP technique for better coverage with enhanced APFD considering the execution time of test cases.	Coverage-based	[25]
2015	Harish *et al*.	Coupling effect-based TCP technique that considers the module coupling effect while prioritizing tests to achieve higher APFD.	Other	[26]
2015	Dusica *et al*.	Multi-perspective regression TCP for time constraint environments for faster fault detection with maximum test case execution with higher failure frequency and cross-functional coverage.	Other	[27]
2015	Jiang *et al*.	Input-based adaptive randomized cost-efficient TCP techniques provide a higher APFD value than ART and GA.	Search-based	[28]
2015	Konsaard *et al*.	Total coverage-based TCP using a modified GA that improves condition coverage and execution time.	Coverage-based	[29]
2015	Noor *et al*.	Similarity-based risk-driven TCP enhances the risk of a test case even if it is not the same as a failed test case but is like a failing test case.	History-Based	[30]
2016	Busjaeger *et al*.	A framework integrates existing TCP techniques through machine learning.	History-based	[31]
2016	Eghbali *et al*.	A TCP approach to increase entity coverage by using the Greedy Algorithm.	Coverage-based	[32]
2016	Marchetto *et al*.	A Source code coverage, requirements coverage, execution time-based TCP technique utilizing non-dominated sorting genetic algorithm II (NSGA-II).	Coverage-based	[33]
2016	Ansari *et al*.	A TCP approach using ACO Algorithm to increase the fault detection rate and reduce cost and time.	Other	[34]
2017	Chen *et al*.	Adoptive random sequence-based TCP that provides early fault detection and more effectiveness than random prioritization TCP techniques.	Other	[28]
2017	Xiao *et al*.	The clustering approach combines fault prediction to enhance the effectiveness of TCP.	Other	[35]
2017	Wang *et al*.	QTEP, a quality-aware TCP that considers fault proneness of code and improves existing coverage-based techniques by utilizing static defect prediction, and static bug-finder.	Coverage-based	[36]
2017	Aggarwal *et al*.	Combinatorial test data set prioritization by using data flow techniques. It provides better fault detection than unordered t-way test cases.	Coverage-based	[37]
2017	Marijan *et al*.	TITAN prioritization is based on a higher fault detection rate, on the failures with high impact on users, higher requirement coverage, and test case execution time.	Coverage-based	[38]
2017	Bian *et al*.	Coverage and execution time-based TCP technique using the ACO algorithm.	Coverage-based	[39]
2017	Hasan *et al*.	A dissimilarity clustering-based TCP technique using historical data.	History-based	[40]
2018	Miranda *et al*.	Scalable similarity-based TCP in both black box and white box fashion.	Similarity-based	[41]
2018	Ozturk *et al*.	A bat-inspired algorithm-based that considers the cost of individual test cases and gives the best complexity percentage of fault detection correlation.	Fault-based	[42]
2018	Abdur *et al*.	Test cases are prioritized based on dissimilarity among test cases.	History-based	[43]
2019	Matinnejad *et al*.	A TCP technique using test coverage of test suit, their output diversity as a representation of fault revealing probability implemented through Greedy Algorithm.	Coverage-based	[44]
2019	Khatibsyarbini *et al*.	A TCP method using similarity and dissimilarity weights and uniqueness of test cases, test cases distance implemented through the Firefly Algorithm.	Similarity-based	[45]
2019	Tahvili *et al*.	sOrTES supportive tool as TCP approach using requirements coverage, execution time, the functional dependency between test cases.	Requirements-based	[46]
2019	Mukherjee *et al*.	A technique using modified lines covered by a test case, execution time, the maximum amount of time required for the execution of a prioritized test case. GA, ACO, Simulated Annealing, and Knapsack Problem are used.	Coverage-based	[47]
2019	Lu *et al*.	An ACO-based TCP method to increase code coverage.	Coverage-based	[48]
2020	Jahan *et al*.	A TCP technique based on system method risk values.	Risk-based	[49]
2020	Lima *et al*.	A Multi-Armed Bandit TCP approach for a continuous integration environment is proposed.	History-based	[50]
2020	Zhou *et al*.	A distance-based TCP approach to beat random test prioritization.	Other	[51]
2020	Mohd- *et al*.	A model-based TCP technique to boost fault detection.	Model-based	[52]
2020	Venugopal *et al*.	A modification-aware TCP technique is proposed.	Modification-based	[53]
2020	WANG *et al*.	A new TCP method for service-oriented web applications using modification information.	Modification-based	[54]
2021	Iqbal *et al*.	A TCP approach for regression testing of model transformations	Model-based	[55]
2021	Cheng *et al*.	A TCP approach for configuration testing.	Coverage-based	[56]
2021	Bagherzadeh et al.	Re-enforcement learning-based TCP is proposed for continuous integration	Other	[57]

The focus is on the number of faults detected instead of their impact on the client’s business. Ignoring value in the TCP process is prone to produce unsatisfactory results. Another problem with this fashion is that the use of value-neutral metrics for performance evaluation of TCP techniques will produce unreliable results. To address these problems, value-based cost-cognizant TCP techniques have been introduced.

The value-based cost-cognizant TCP techniques deal with the severity of faults and the cost of test cases in the prioritization process [58]. The value-based TCP takes the challenge of integrating value consideration into the prioritization process. The value orientation in TCP ensures that prioritization satisfies its value objectives. In practice, 80% of the value exists in a 20% portion of the software [3, 8]. This fact supports the need for value-orientation in software testing. Unfortunately, a limited number of value-based cost-cognizant TCP techniques are available in the literature. To the best of the author’s knowledge, no comprehensive review is available on value-based TCP techniques. In this paper, we have performed a systematic literature review of value-based cost-cognizant TCP techniques to know the current state of research in this domain. An enhanced taxonomy of TCP techniques has been proposed so that value considerations can be taken into account in the TCP process. A generic cost-cognizant TCP [59–61] process with its objectives and an analysis of the proportional differences of value-neutral and value-based TCP techniques are also given. This study emphasized the need for value-orientation in TCP and highlighted that a paradigm shift is required from a value-neutral to value-based TCP process. The study results yield that there is very lesser work done in value-based TCP as compared to value-neutral TCP. The study also highlighted a few open research problems and concluded that there is a great potential for further research in value-based TCP. To perform the study, standard SLR guidelines recommended by Kitcheham have been followed [62].

## 2. Method

**Fig 2:**
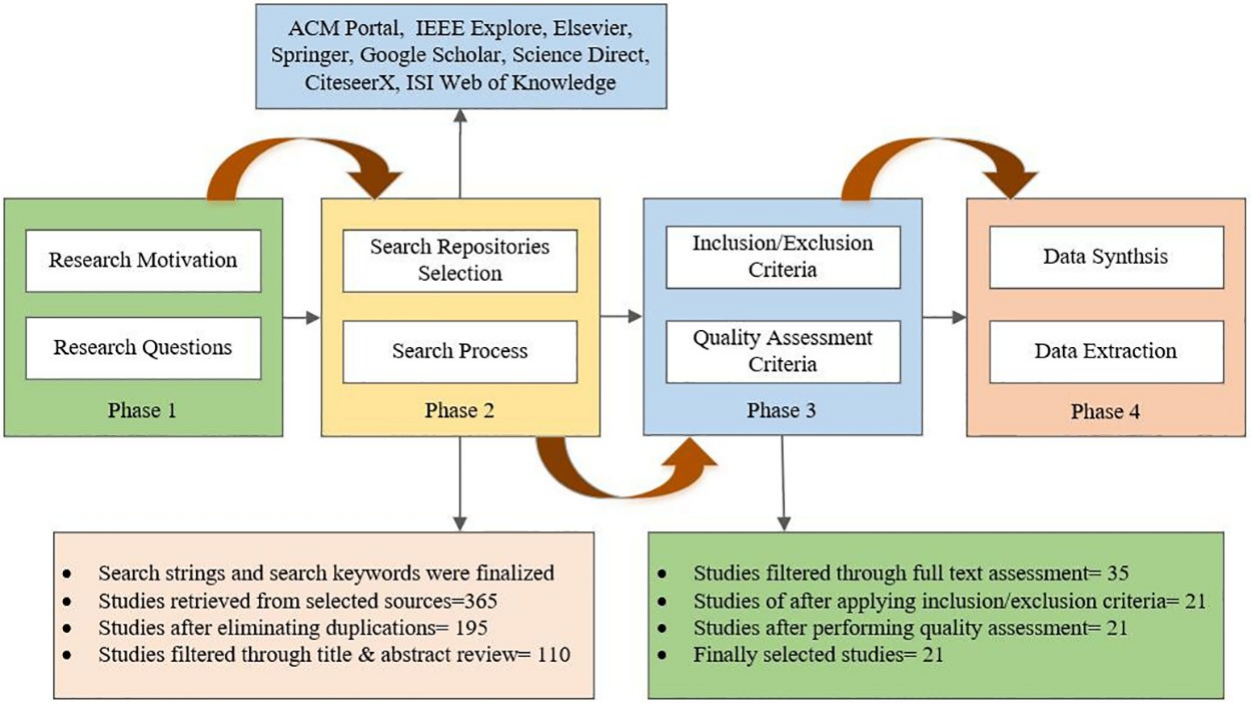
Review protocol phases.

This study has been undertaken as an SLR following the standard guidelines proposed by Kitchenham and Carter [62, 63]. An SLR is a great means to know the status of research related to a specific phenomenon or a particular domain. Hence, the goal of this SLR is, to sum up the knowledge related to value-based cost cognizant TCP techniques. The review protocol for this study contains four phases, each with two steps. In the first phase, research motivation and research questions have been described. The second phase is related to the selection of search repositories and the search process. The third phase describes two study selection criteria, including inclusion/exclusion criteria and quality assessment criteria. The last phase is related to data synthesis and data extraction. An external reviewer performed evaluation and validation of the review protocol and provided feedback. All the feedback suggestions are incorporated to refine and improve the overall quality of the protocol. The review protocol is shown in Fig 2.

### 2.1 Research motivation

Different SLRs have been published on different aspects of TCP. In [64], an SLR of TCP approaches for regression testing has been performed by Khatibsyarbinni *et al*. The goal of this SLR is to comprehend the current trend of TCP approaches and to provide their empirical evaluation. The taxonomic distribution of the TCP approaches is presented. It covers the strengths and weaknesses of TCP approaches in terms of their results. It also covered the processes and artifacts involved in TCP and metrics used for the evaluation of TCP techniques. In [65], a survey is conducted on TCP techniques which contains a description of cost-cognizant TCP techniques. As per this study, Malishevsky *et al*. has suggested the cost cognitive metric APFDc for the performance evaluation of TCP techniques. APFDc considers varying test cases cost and fault severity. This new metric is proposed to address the limitations of the existing metric APFD. It accounts for units of fault severity detected by units of test case cost. According to a study [64], the average percentage of fault detection (APFD) 51%, coverage effectiveness (CE) 10%, APFDc 9%, Execution Time (ET) 7%, and other metrics have been used 23% for the performance evaluation of TCP techniques. A mapping study highlighted the major categories of TCP techniques including coverage-based, distribution-based, requirements-based, model-based, human-based, probabilistic-based, history-based, cost-aware-based, and others [58].

According to a study [66], the usage of performance metrics is 58% (APFD), 8% (APFDc), 8% (APSC), 6% (NAPFD), and others 2%. In [17], mapping study results have been presented related to the test prioritization in a continuous integration environment. In [67], a review of TCP techniques using GAs is done that covers methodologies, adequacy criteria, algorithms, dataset specifications, performance evaluation metrics, and validation criteria. According to this study, different metrics have been used like Execution time (ET) 48%, APFDc 18%, Expense 15%, Fault Detection 33%, APFD 24%, and NAPFD 9%. Another mapping study indicates that the usage of APFD is on top, APFDc on second and APSC is the least used metric [68].

The literature describes cost-aware/cost-cognizant as an explicit category of TCP techniques, but there is no SLR available on it. The mainly used TCP evaluation metrics are APFD and APFDc. For value-based cost-cognitive TCP techniques, the APFD metric is not appropriate because it has two limitations a) all test cases have equal cost and b) all faults have equal severity [69]. The use of the APFD metric for performance evaluation of value-based TCP techniques is prone to produce unsatisfactory results. These research gaps found in existing studies are the major motivation and inspiration that raised a need to publish a technically informative document in the domain of value-based-cost-cognizant TCP techniques. To fill this research gap, an SLR of value-based cost-cognizant TCP techniques is performed in this study. Its objective is, to sum up, the knowledge related to value-based cost-cognizant TCP techniques and to highlight the related open research problems of this domain.

To execute the SLR, a review protocol is developed to control the researcher’s bias. It consists of research questions, selection of the literature sources, search process, study selection procedure, quality assessment score, and data extraction and data synthesis. The review is assessed and validated by an external reviewer. Few suggestions are received and are incorporated to improve its quality. Each step of the review protocol is comprehensively described below.

### 2.2 Research questions

Six research questions have been articulated that are required to be answered through this research. These questions are listed in Table 2. The motivation behind each research question is also presented.

**Table 2:** Research questions.

RQ	Question	Motivation
RQ1	Which algorithms are used in value-based cost-cognizant TCP?	To know the state-of-the-art algorithms used for the implementation of the value-based TCP technique.
RQ2	Which methods (e.g. Empirical study, Experiment, Case study, and Industrial case study) are used for results validation of value-based cost-cognizant TCP?	To know the common methods of results validation for value-based cost cognizant TCP techniques.
RQ3	What are the generic steps of the value-based cost cognizant TCP process and its objectives?	To know the common procedure of value-based TCP techniques.
RQ4	What is the enhanced taxonomy of TCP techniques considering value?	To know the current value-based categorization of TCP techniques in a taxonomic form.
RQ5	Which metrics are used for the performance evaluation of value-based TCP techniques?	To distinguish value-based metrics from value-neutral metrics.
RQ6	What are the open research problems related to TCP and Recommendations to Fill the Research Gaps?	To highlight the limitations of current trends in TCP and to provide suggestions to fill the current research gaps.

To define the scope of selected studies and goals of this SLR we used the Population, Intervention, Comparison, Outcomes, and Context (PICOC) method [67] as mentioned below. It helped us to reduce the risk of bias.

***Population***: *Literature on value-based cost-cognizant TCP*.
***Intervention***: *Taxonomic classification of TCP*.
***Comparison***: *Comparison among interventions to analyze current research of different methods*.
***Outcomes***: *Recommendations for further research on value-based TCP for a paradigm shift with evidence*.
***Context***: *An SLR to combine the current body of knowledge*.

### 2.3 Selection of literature sources

The selection of the literature sources is an important step for any SLR. We selected the popular research repositories that are evident to report research papers related to TCP. The other justification behind the selected research repositories is that few existing reviews of TCP also utilized the same sources [17, 64, 67]. The following database repositories are utilized for this SLR.

- ACM Portal
- IEEE Explore
- Elsevier
- Springer
- Google Scholar
- Science Direct
- CiteseerX
- ISI Web of Knowledge
- IEEE Computer Society

### 2.4 Search process

The search strings were formulated considering the research questions and study goals. The search strings were composed of the terms “Test Case Prioritization”, “Value-Based TCP”, “Cost-Cognizant TCP”, and “Evaluation Metrics for TCP”. The keywords used in the search process are listed below.

- Test case prioritization
- Value-based test case prioritization
- Cost-aware test case prioritization
- Cost-cognizant test case prioritization
- Test case prioritization reviews
- Test case prioritization for regression testing
- Evaluation metrics for test case prioritization

According to our search strategy, the above search strings are applied to the selected literature source databases. The literature is extracted from 2001 to August 2021. No paper was found before 2001. Total 365 papers are retrieved. ACM Portal returned 45 papers, IEEE Explore 52, Elsevier 55, Springer 20, Google Scholar 34, Science Direct 32, CiteseerX 32, ISI Web of Knowledge 30, and IEEE Computer Society returned 65 papers.

### 2.5 Study selection procedure

**Fig 3:**
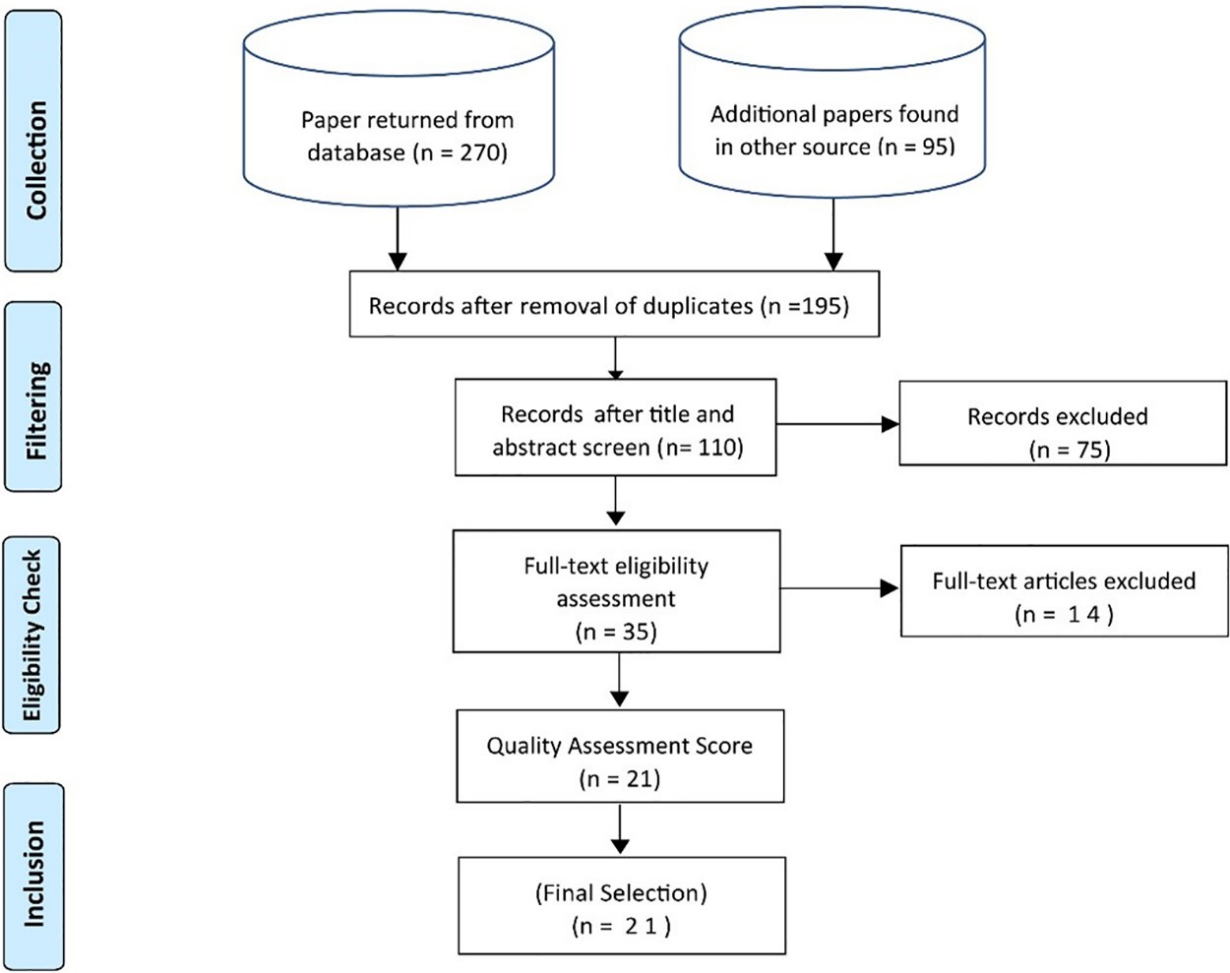
PRISMA flow diagram for search process and selection procedure.

The study selection procedure consisted of a set of steps, presented in Fig 3 following the PRISMA (Preferred Reporting Items for Systematic reviews and Meta-Analyses) statement [70]. A proper study selection procedure is adopted to select relevant studies and remove all irrelevant studies. An inclusion and exclusion criteria are defined to ensure that only relevant studies are selected for the study. Inclusion and exclusion criteria are presented in Table 3. The primary author selected primary studies. A test/retest approach is opted to verify the selection process. The co-author (Ph. D. Research Supervisor) compared the results using random sampling. The appropriateness of inclusion and exclusion criteria is tested and verified as per the guidelines of Brereton et al. [71]. The inclusion and exclusion criteria facilitate the selection of studies to be considered for SLR and it is utilized by existing reviews of TCP [64, 67]. The opted inclusion/exclusion criteria for this SLR are presented in Table 3.

**Table 3:** Inclusion and exclusion criteria.

Inclusion Criteria	Exclusion Criteria
Papers on cost-aware/cost cognizant TCP techniques	Papers not related to cost-aware/ cost-cognizant TCP techniques
Papers on value-based TCP techniques	Papers with no value-orientation
Papers on evaluation metrics/measures for cost-cognizant TCP	Papers without evaluation metrics and empirical study
The papers that are written in the English language only	Papers that are presented in a language other than English
The latest revised version of the papers is considered	Papers with duplicate revised versions have been removed

### 2.6 Quality assessment score

The Quality Assessment Score (QAS) provides help to evaluate the relevance and significance of the study [63]. A study is selected or rejected based on the QAS. For study selection, we formulated a three-point QAS checklist following the guidelines of Kitchenham et al. [63]. The checklist is given in Table 4.

**Table 4:** QAS checklist.

S. No.	QAS Question	Feedback	QAS
1.	Does the study propose a value-based cost-cognizant TCP technique?	(Yes = 2) (Partial = 1) (No = 0)	
2.	Does the study contain empirical evaluation?	(Yes = 2) (Partial = 1) (No = 0)	
3.	Does the study use cost cognizant evaluation metrics?	(Yes = 2) (Partial = 1) (No = 0)	

Based on the defined search process, we retrieved 365 papers from the selected literature sources. First, 170 duplicate papers were removed. On the remaining 195 papers, the title and abstract review were performed, and 75 papers were screened out. Through a detailed review of the full text and by applying inclusion and exclusion criteria, 14 more studies were removed. Afterward, we performed a quality assessment and as a result, 14 more studies were removed. Finally, 21 papers were selected for the study. The search process and selection procedure are depicted in PRISMA flow diagram in Fig 3.

Table 5 presents the list of selected studies along with their QAS. The final acceptance criteria for minimum QAS value was “4”. All 21 papers qualified minimum acceptance criteria. To justify the range of literature years covered in this paper, it is mentioned here that we also reviewed the latest papers of 2021, and Table 1 contains multiple papers of 2020 and 2021. The selected studies in Tables 5 and 7 do not include any paper of years 2020, and 2021 because they do not meet the established inclusion criteria. That is the reason for not including any paper of 2020, and 2021 in the selected studies.

**Table 5:** Summary of selected studies.

P. ID	Author	Contribution	QAS
P1	Yu *et al*. (2019), [72]	An approach TERMINATOR for the prioritization of automated UI test cases. There is a computational overhead it recursively updates the Support Vector Machine (SVM) model to tweak the order of un-executed tests. Increased fault detection without the availability of source code. Made dataset available to reproduce results.	6
P2	Ashraf *et al*. (2017), [73]	A value-based TCP technique based on six prioritization factors using PSO to enhance the fault detection rate. An experimental method is used to validate the results.	4
P3	Miranda *et al*. (2017), [74]	A scope-aided TCP method for a better fault detection rate.	5
P4	Wang *et al*. (2015), [75]	A faults-severity-based TCP method to increase fault detection by accumulative severity detected by a test case.	5
P5	Epitropakis *et al*. (2015), [76]	An empirical evaluation of seven algorithms has been done on their fault detection capability and maximizing coverage. APFDc is used as an evaluation metric.	6
P6	Rauf *et al*. (2015), [77]	A value-based TCP method using PSO algorithm to enhance the fault detection rate. An experiment is done to prove the results.	4
P7	Hoq *et al*. (2015), [78]	A dependency cognizant TCP technique to detect more severe faults earlier in the testing life cycle within minimum test case execution time.	5
P8	Yc *et al*. (2012), [59]	A history-based cost-cognizant TCP method by applying a GA to produce an effective test case order. A controlled experiment is performed to validate the results.	5
P9	Li *et al*. (2013), [79]	A value-based prioritization method that lets tests be ordered by how well the tests can decrease risk exposure. Combining this with the tests’ relative costs aids them to be prioritized in terms of the Return on Investment (ROI) or risk reduction leverage (RRL). A novel metric Average Percentage of Business Importance Earned (APBIE) is proposed for performance evaluation.	5
P10	Marijan *et al*. (2013), [80]	ROCKET a TCP approach for continuous regression testing, was applied to an industrial case study to increase the fault detection rate with minimum execution time.	5
P11	Ashraf *et al*. (2012), [81]	A TCP algorithm that orders the system test cases based on the six different factors: customer priority, changes in the requirement, requirement traceability, execution time, implementation complexity, and fault impact of the requirement.	4
P12	Ramler *et al*. (2012), [9]	A value-based coverage approach for requirement-based testing to enhance business value coverage.	4
P13	Zhang *et al*. (2011), [82]	A new cost cognizant metric is proposed for the performance evaluation of TCP techniques.	4
P14	Bryce *et al*. (2011), [83]	A cost-based combinatorial interaction coverage TCP technique and a new metric for it are proposed. An improvement in fault detection is evident through an empirical study.	5
P15	Askarunisa *et al*. (2010), [19]	Cost and Coverage based TCP technique is proposed and cost and coverage-based metrics are used for performance evaluation.	5
P16	Park *et al*. (2008), [61]	A value-based cost cognizant TCP approach based on historical value to estimate fault severity and test cases cost to improve regression testing effectiveness.	5
P17	Zhang *et al*. (2007), [84]	A cost-cognizant TCP method based on varying requirements priority and test cases cost. A new evaluation metric MRP_TC has also been proposed.	4
P18	Malishevsky *et al*. (2006), [60]	The cost-cognizant TCP method and a new metric cost-cognizant evaluation metric APFDc that consider fault severity and test case cost in the TCP process.	6
P19	Srikanth *et al*. (2005), [85]	A value-based TCP approach based on the Prioritization of Requirements for Tests (PORT) is presented. A case study was done to prove the results and an increase in severe faults detection is evident.	5
P20	Srikanth *et al*. (2005), [86]	A requirements-based TCP technique to boost the rate of detection of severe faults.	5
P21	Elbaum *et al*. (2001), [87]	A new evaluation metric that incorporates varying test cases cost and fault severity for cost-cognizant TCP.	6

### 2.7 Data extraction and data synthesis

In any review protocol, the process of extracting, and synthesizing data from the selected studies is a prominent reason that distinguishes an SLR from a traditional literature review. In the extraction process, data is extracted from the selected studies relevant to the SLR questions whereas the data synthesis process is the collective form of the results derived from those studies [62]. In the data extraction process, we collected bibliographic information (Unique ID, title, authors, year of publication, citations, paper type, publisher), common steps in the TCP process, the algorithm used, evaluation metrics used, the results validation method, the dataset availability, contribution, category of TCP technique, and open research problems. To collect data from the primary studies, a data extraction form is designed and is given in Table 6. In the data synthesis process, the extracted data is combined and organized in such a way that it can be useful to answer the defined research questions. Table 6 presents the outcome of the data synthesis process.

**Table 6:** Data extraction form.

S. No.	Characteristic	Value
1.	Algorithm used	• TERMINATOR Algorithm
		• Particle swarm optimization (PSO)
		• Additional Greedy
		• Sorting Algorithm
		• Genetic Algorithm
		• Custom algorithm
		• Total Statement Coverage
		• Function coverage
		• PORT Algorithm
2.	Evaluation metric used	• APFDc
		• APFDc
		• APBIE
		• APFDa
		• MRP_TC
		• ASFD
3.	Results validation method used	• Empirical study
		• Experiment
		• Case study
		• Industrial case study
4.	Dataset availability	• Yes
		• No
5.	Category	• History-based
		• Search-based
		• overage-based
		• Fault-based
		• Requirements-based
		• Risk-based

## 3. Data extraction results

Table 7 presents the data extraction results from the selected studies.

**Table 7:** Data extraction results.

P.ID	Author	Prioritization Algorithm Used	Evaluation Metric Used	Results Validation	Dataset Availability	Category
P1	Yu *et al*. (2019), [72]	TERMINATOR Algorithm	APFDc	Empirical study	Yes	History-based
P2	Ashraf *et al*. (2017), [73]	Particle swarm optimization PSO	APFD	Experiment	No	Search-based
P3	Miranda *et al*. (2017), [74]	Additional Greedy	APFDc	Experiment	No	Coverage based
P4	Wang *et al*. (2015), [75]	Sorting Algorithm	APFD	Experiment	No	Fault-based
P5	Epitropakis *et al*. (2015), [76]	-------	APFDc	Empirical study	No	Coverage-based
P6	Rauf *et al*. (2015), [77]	Particle swarm optimization PSO	APFD	Experiment	No	Fault-based
P7	Hoq *et al*. (2015), [78]	Sorting Algorithm	APFDc	Experiment	No	Fault-based
P8	Yc *et al*. (2012), [59]	Genetic Algorithm	APFDc	Experiment	No	History-based
P9	Li *et al*. (2013), [79]	-------	APBIE	Industrial Case study	No	Coverage-based
P10	Marijan *et al*. (2013), [80]	-------	APFDc	Industrial Case study	No	Fault-based
P11	Ashraf *et al*. (2012), [81]	Particle swarm optimization PSO	APFD	Experiment	No	Search-based
P12	Ramler *et al*. (2012), [9]	-------	-------	-----	No	Coverage-based
P13	Zhang *et al*. (2011), [82]	-------	APFDa	Case study	No	Fault-based
P14	Bryce *et al*. (2011), [83]	Custom algorithm	APFDc	Empirical study	No	Coverage-based
P15	Askarunisa *et al*. (2010), [19]	Total Statement Coverage	APFDc	Experiment	No	Coverage- based
P16	Park *et al*. (2008), [61]	------	APFDc	Experiment	No	History-based
P17	Zhang *et al*. (2007), [84]	------	MRP_TC	Case Study	No	Requirements-based
P18	Malishevsky *et al*. (2006), [60]	Function coverage	APFDc	Case study	No	Fault-based
P19	Srikanth *et al*. (2005), [85]	PORT Algorithm	ASFD	Case study	No	Requirements-based
P20	Srikanth *et al*. (2005), [86]	------	ASFD	Experiment	No	Requirements-based
P21	Elbaum *et al*. (2001), [87]	------	APFDc	Case study	No	Fault-based

## 4. Assessment and findings

After a comprehensive analysis of the selected studies and synthesized data, the assessment is performed, and the findings are concluded. In this section, all the defined research questions have been answered. The first paper related to value-based TCP published in 2001, proposed a cost-cognizant performance evaluation metric APFDc [87]. Later, few other authors used this metric for the performance evaluation of their proposed TCP technique [19, 59–61, 72, 74, 76, 78, 80, 83]. We found that only 21 papers are published in a value-based fashion which used test case cost and fault severity in the test case prioritization process. Out of 21 selected papers, there are 4 conference papers, and the rest of the seventeen are published in different journals. Table 8 presents the authors, year of publication, reference, and publisher information. The current trend of value-based cost-cognizant TCP techniques shows that limited work is available in a value-based fashion. Therefore, more TCP studies are required in a value-based fashion to get better and reliable results. There is a great potential for further research to fill the gaps. The leading researchers in the domain of value-based cost-cognizant TCP techniques are Gregg Rothermel, Sebastian Elbaum, and Alexey Malishevsky [60, 87].

**Table 8:** Research trends of value-based cost-cognizant TCP techniques.

P.ID	Author	Year	Reference	Publisher
P1	Yu *et al*.	2019	[72]	Journal
P2	Ashraf *et al*.	2017	[73]	Journal
P3	Miranda *et al*.	2017	[74]	Journal
P4	Wang *et al*.	2015	[75]	Journal
P5	Epitropakis *et al*.	2015	[76]	Journal
P6	Rauf *et al*.	2015	[77]	Journal
P7	Hoq *et al*.	2015	[78]	Conference
P8	Yc *et al*.	2012	[59]	Journal
P9	Li *et al*.	2013	[79]	Journal
P10	Marijan *et al*.	2013	[80]	Conference
P11	Ashraf *et al*.	2012	[81]	Journal
P12	Ramler *et al*.	2012	[9]	Journal
P13	Zhang *et al*.	2011	[82]	Conference
P14	Bryce *et al*.	2011	[83]	Journal
P15	Askarunisa *et al*.	2010	[19]	Journal
P16	Park *et al*.	2008	[61]	Conference
P17	Zhang *et al*.	2007	[84]	Journal
P18	Malishevsky *et al*.	2006	[60]	Journal
P19	Srikanth *et al*.	2005	[85]	Journal
P20	Srikanth *et al*.	2005	[86]	Journal
P21	Elbaum *et al*.	2001	[87]	Journal

### 4.1 Algorithms used in value-based cost-cognizant TCP (RQ1)

The synthesized data extracted from the selected studies show that different algorithms have been used in value-based cost-cognizant TCP techniques. The algorithms include TERMINATOR Algorithm, Particle swarm optimization (PSO), Additional Greedy, Sorting Algorithm, Genetic Algorithm, Custom algorithm, Total Statement Coverage, Function coverage, and PORT Algorithm. Nine studies did not use any algorithm because they sorted their test cases based on some prioritization criteria. PSO is a dominantly used algorithm by the studies P2, P6, and P11.

**Fig 4:**
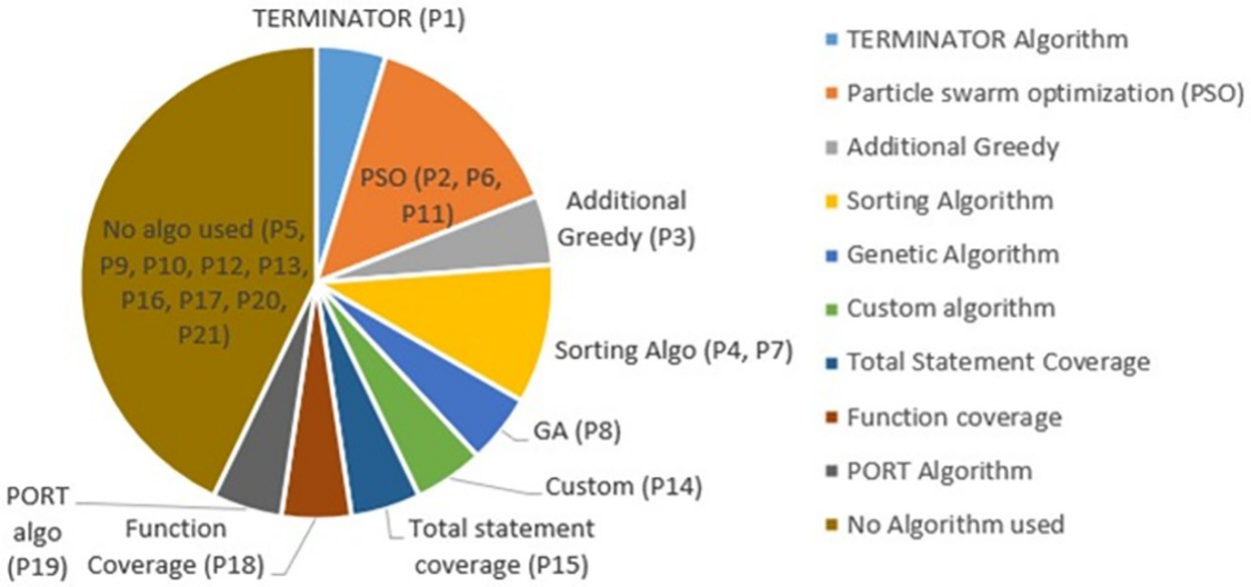
Distribution of studies according to the algorithm used.

Fig 4 shows study distribution according to the algorithm used in the selected studies.

### 4.2 Validation methods used in value-based cost-cognizant TCP (RQ2)

**Fig 5:**
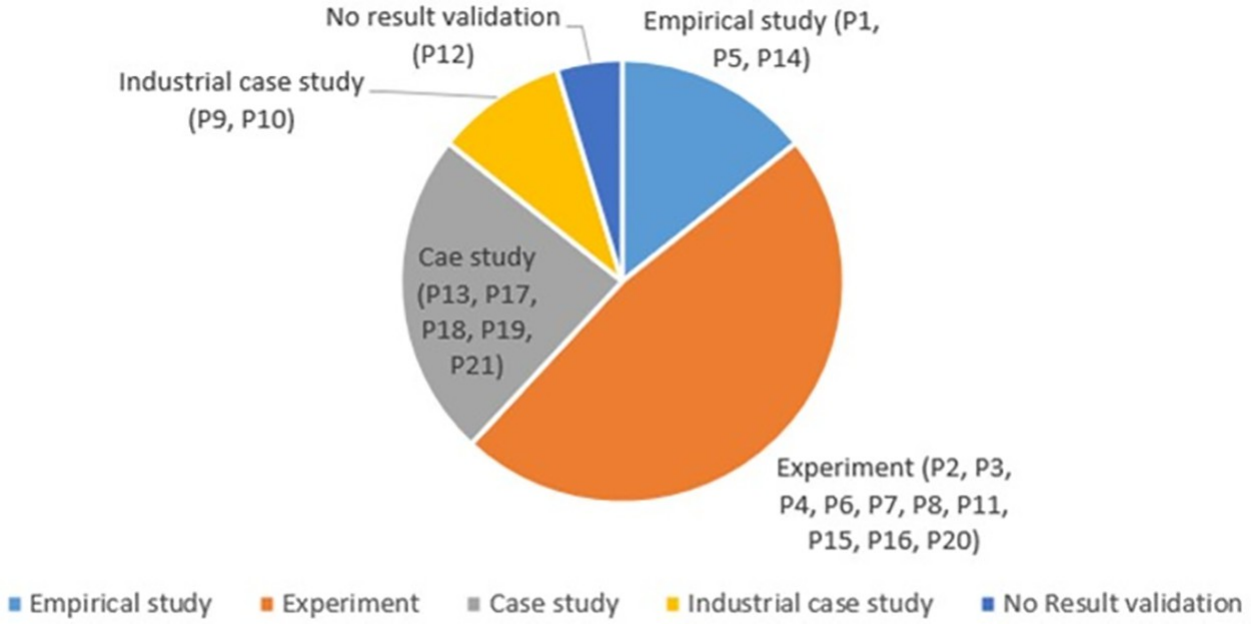
Distribution of studies according to results validation method used.

The collective results extracted from the selected studies indicate that four results validation methods have been used including, Empirical study, Experiment, Case study, and Industrial case study. Papers P1, P5, and P14 used the empirical study method to validate their results. Empirical evaluation is usually based on the researcher’s observations and investigation of the phenomenon. Papers P2, P3, P4, P6, P7, P8, P11, P15, P16, and P20 used the experiment method to compare results with the existing state-of-the-art techniques. The experiment method is usually applied for a small project. Most of the studies are done with a small scope. Papers P13, P17, P18, P19, and P21 used the case study method to validate their results. The case study is usually applied to a specific case to validate the results. Papers P9 and P10 used the industrial case study method to validate the results. Industrial case studies usually cover real industrial projects. Paper P12 did not use any validation method. Fig 5 shows the distribution of selected studies according to the validation method used.

### 4.3 Value-based cost-cognizant TCP process and its objectives (RQ3)

**Fig 6:**
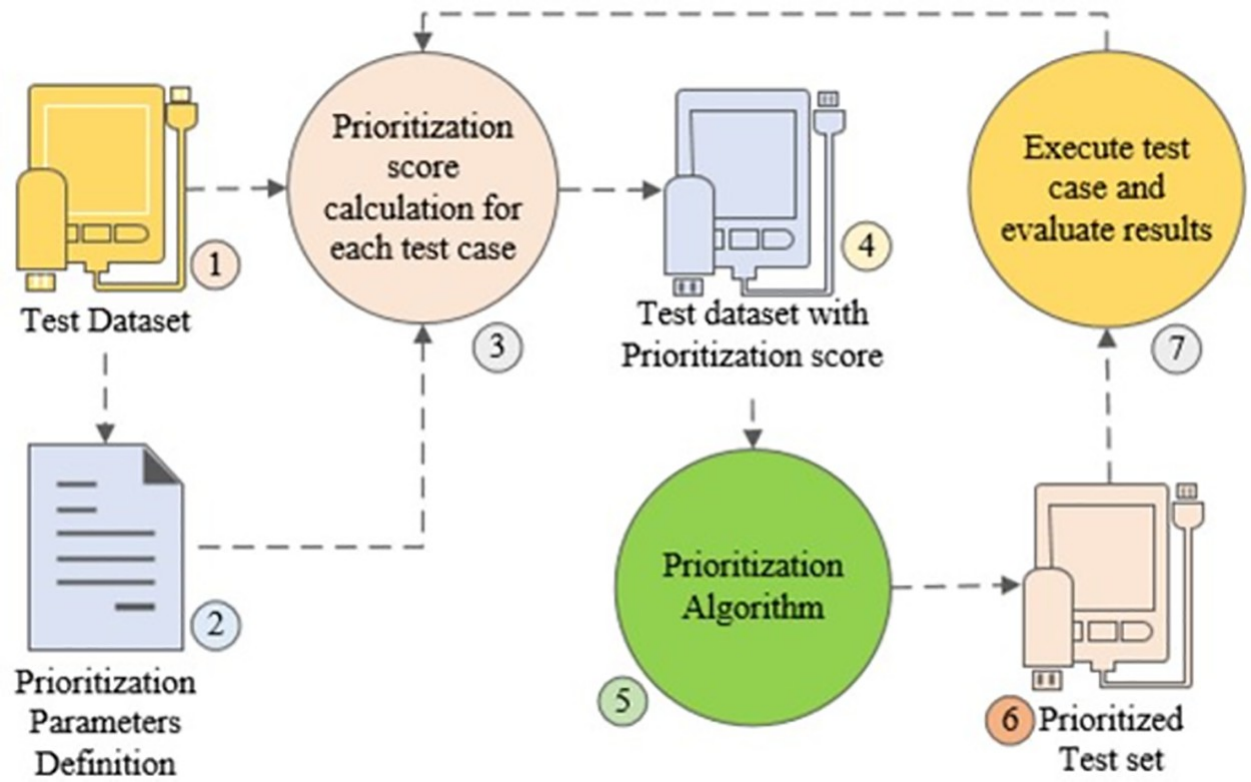
Test case prioritization process.

The use of standard processes and clarity of objectives are vital for the success of software projects. In the TCP process, test cases are not eliminated or removed, rather each test case is assigned a priority. The test cases in the test suite are sorted by their priority. Then the testing team starts executing the test cases with the highest priority and ends when regression time ends, or all test cases are covered. A variety of TCP techniques are available including search-based, requirements-based, coverage-based, history-based, fault-based, risk-based, and others [64]. This sub-section outlines a TCP process that depicts some common steps involved in value-based cost-cognizant TCP techniques. These steps are taken from the selected studies [61, 73, 81] and are depicted in Fig 6.

1. *Prepare the test case data set that needs to be prioritized*.
2. *Define the parameters to be used for prioritization assuming that different faults may have different severity and different test cases may have a different cost*.
3. *Apply formula to calculate the prioritization score for each test case*.
4. *Prepare test data set with prioritization score that needs to be prioritized*.
5. *Run the algorithm to prioritize the test cases based on prioritization criteria/score*.
6. *Prepare test dataset in prioritized order*.
7. *Execute the test cases as per assigned priority in the above step using a value-based cost-cognizant metric and evaluate the results*. *Value-based cost cognizant metric is a metric in which varying severity of faults and test cases cost is considered*.
8. *Repeat step 3*.

**Fig 7:**
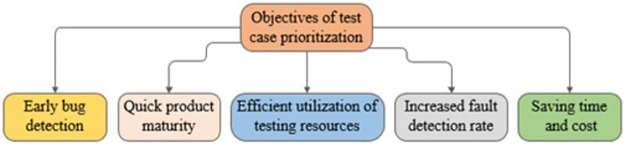
The objectives of test case prioritization.

Few common objectives of value-based cost-cognizant TCP techniques given in selected studies have also been presented and are depicted in Fig 7.

- Early fault severity detection is the major objective of value-based TCP. Late detection of bugs is more costly. Therefore, there is a direct relationship of the cost with this TCP objective.
- To provide quick product maturity through critical fault detection first. Maximum bugs are detected and fixed earlier therefore product gets mature and it ultimately builds confidence to meet the deadline.
- Efficient utilization of testing resources during regression testing.
- Early business value coverage
- Saving time and budget

### 4.4 An enhanced taxonomy of TCP techniques (RQ4)

This section presents the details about the categorization of TCP techniques given in the selected studies. According to a study, there are seven categories of TCP techniques including search-based, coverage-based, requirements-based, fault-based, risk-based, history-based, and others [64]. The most widely used approach is Search-based, while other techniques used are coverage-based and fault-based. Another study presented a categorization including probabilistic, cost-based, history-based, human-based, distribution-based, coverage-based, model-based, and others [17]. Table 9 shows the categorization of value-based cost-cognizant TCP techniques selected for this study.

**Table 9:** Classification of value-based cost-cognizant TCP techniques.

Categories	Paper ID	References	Year of Publication
Search-based	P2, P11	[73, 81]	2017, 2012
Coverage-based	P5, P9, P12, P14, P15,	[19, 65, 76, 79, 83]	2015, 2013, 2012, 2011, 2010,
Requirement-based	P3, P17, P19, P20	[74, 84–86]	2017, 2007, 2005, 2005
Fault-based	P4, P6, P7, P10, P13, P18, P21	[60, 75, 77, 78, 80, 82, 87]	2015, 2015, 2015, 2013, 2011, 2006, 2001
History-based	P1, P8, P16	[59, 61, 72]	2019, 2012, 2008

**Fig 8:**
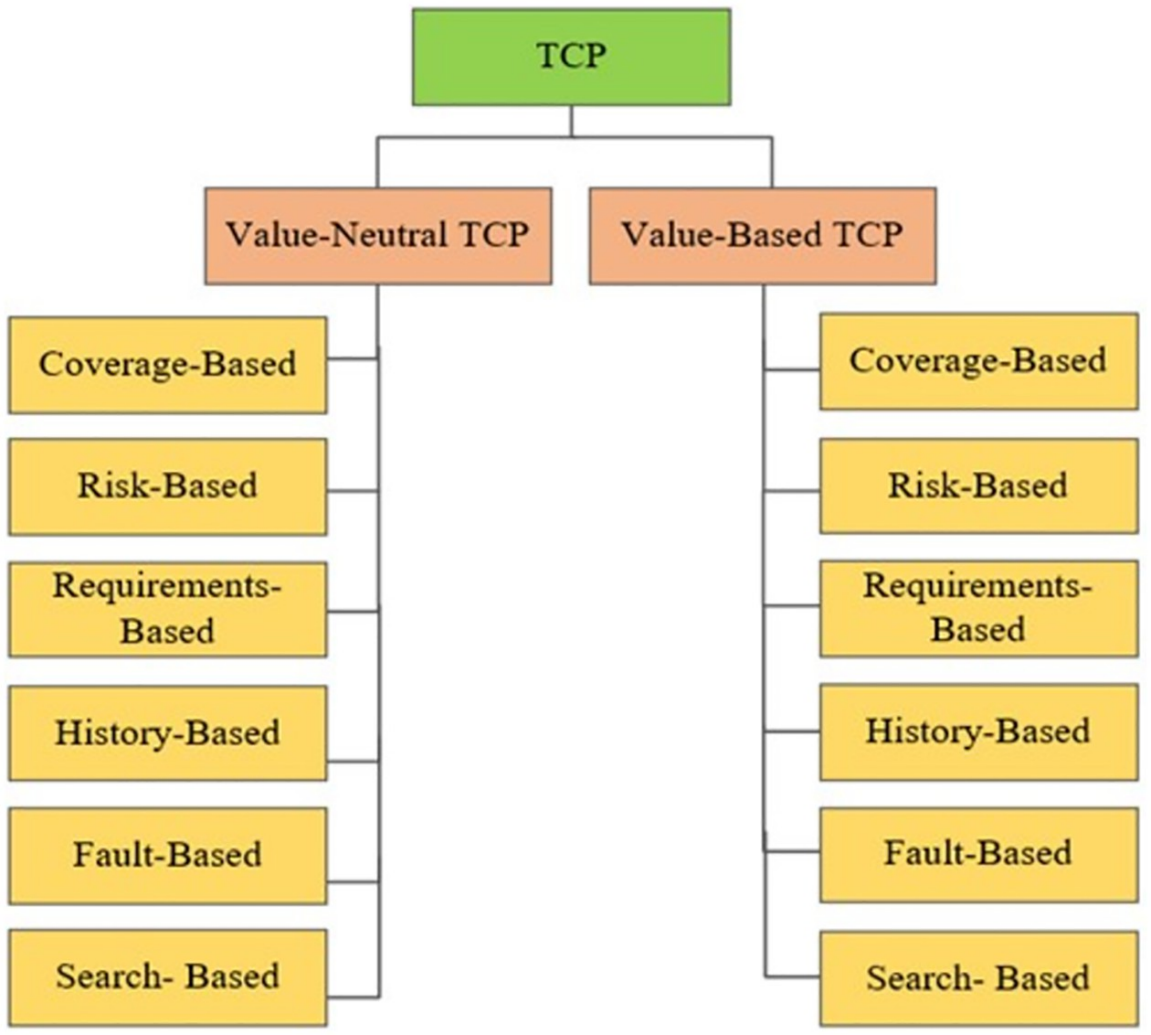
Enhanced taxonomy of test case prioritization.

The existing categorizations and taxonomies of TCP techniques have recognized “cost-based” as one category among other categories. But we believe that “cost-based” or “cost-cognition” is a value-based fashion. We must recognize cost-cognizant test prioritization to segregate it from value-neutral TCP techniques. To address this need we proposed two abstract classes of TCP techniques including value-neutral and value-based. For this, we have proposed an enhanced taxonomy of TCP techniques presented in Fig 8. Our enhanced taxonomy of TCP techniques is comprised of two major classes including value-neutral test prioritization and value-based test prioritization.

### 4.5 Performance evaluation metrics used in cost-cognizant TCP (RQ5)

For the validation of any proposed technique, its performance is evaluated. This section presents the performance evaluation metrics used for TCP techniques given in the selected studies. There are many metrics used for the performance evaluation of TCP techniques. Table 10 shows the list of metrics used in value-based cost-cognizant TCP techniques mentioned in the selected studies.

**Table 10:** Performance evaluation metrics used in cost-cognizant TCP.

S. No.	Metric/Measure	Metric Description	Reference
1.	$\text{APFD}=1-[\frac{{TF}_{1}+{TF}_{2}+{TF}_{3}+\cdots +{TF}_{m}\;}{mn}]\text{+}\frac{\text{1}}{\text{2n}}$	The average percentage of fault detection	P2, P4, P6, P11
2.	${APFD}_{C}=\frac{\sum_{i=1}^{m}(f_{i}\times (\sum_{j={TF}_{i}}^{n}t_{j}\;-\;\frac{1}{2}\;t_{{TF}_{i}}))}{\sum_{i=1}^{n}t_{i}\times \;\sum_{i=1}^{m}f_{i}}$	The cost-cognizant weighted average percentage of fault detection	P1, P3, P5, P7, P8, P10, P14, P15, P16, P18, P21
3.	${APFD}_{a}=(1-\sum_{i=1}^{m}\frac{\sum_{j=1}^{{TF}_{i}}C_{j}}{m\sum_{j=1}^{n}C_{j}})\;\times \;100\%$	Improved Average percentage of fault detection rate. (Considering the cost of test cases)	P13
4.	${ASFD}_{i}=[\frac{\sum_{j=1}^{m}{SV}_{j}}{TSFD}]$	The average severity of faults detected	P19, P20
5.	$APBIE=\sum_{i=1}^{n}{PBIE}_{i}/n\;$	The average percentage of business importance earned	P9
6.	$M_{RP\_TC}=\frac{\sum_{j=1}^{m}\left(rf_{i}\;\times \;(\sum_{i={TR}_{i}}^{n}{tc}_{i}\;-\frac{1}{2}{\;*\;tcTR}_{i})\right)}{\sum_{j=1}^{m}{rp}_{i\;\times \;\sum_{j=1}^{n}{tc}_{j}}}$	Metric based on Varying Requirement Priority and Test Case Cost	P17
7.	No metric used	No metric	P12

#### 4.5.1 The average percentage of fault detection (APFD)

APFD is the most popular metric used for the performance evaluation of TCP techniques and it was created by Elbaum *et al*. in 2000 [88]. This is a value-neutral metric based on the assumption that all faults have the same severity, and all test cases have the same cost. APFD is presented by Eq 1.


$$\text{APFD}=1-\left[\frac{{\text{TF}}_{1}+{\text{TF}}_{2}+{\text{TF}}_{3}+\cdots +{\text{TF}}_{\text{m}}\;}{\text{mn}}\right]\text{+}\frac{\text{1}}{\text{2n}}$$ (1)

In this formula, m is the total number of faults detected and n is the total number of test cases, and TF1 is the place of the first test case that reveals fault F1.

#### 4.5.2 The average percentage of fault detection per cost (APFD_C_)

APFDc metric was proposed by the researchers to overcome the shortcomings of the APFD metric [87]. APFDc considers varying test cases cost and fault severity. This new cost-cognizant metric was proposed in a value-based fashion. It accounts for units of fault severity exposed by units of test case cost. The x-axis shows the total units of test cases cost instead of simply showing the percentage of test cases executed and similarly y-axis implies the total units of fault severity instead of simply showing the percentage of faults detected. APFDc has been presented by Eq 2.


$$APFD_{C}=\frac{\sum_{i=1}^{m}(f_{i}\times (\sum_{j=TF_{i}}^{n}t_{j}-\frac{1}{2}\;t_{TF_{i}}))}{\sum_{i=1}^{n}t_{i}\times \;\sum_{i=1}^{m}f_{i}}$$ (2)

In Eq 2, *T* is the test suite and *n* is the number of test cases with costs *t1*, *t2*, *…*, *tn*. *F* is a set with *m* number of faults detected by *T*, and *f1*, *f2…*, *fm* are the severities of faults. *TFi* is the first test case in a test case order that detects fault *i*.

#### 4.5.3 The average percentage of fault detection (APFD_a_)

This APFDa is recommended by Zhang *et al* and is an improved form of APFDc [89]. It best describes the physical explanation of the testing process. This metric has been presented by Eq 3.


$$APFD_{a}=\left(1-\sum_{i=1}^{m}\frac{\sum_{j=1}^{TF_{i}}C_{j}}{m\;\sum_{j=1}^{n}C_{j}}\right)\times 100\%$$ (3)

#### 4.5.4 Metric based on varying requirements’ priority and test cases’ cost (*M*_*RP_TC*_)

This metric was introduced by Zhang *et al*. and it is based on varying requirements’ priority and test case cost [84]. It can be represented by Eq 4. The value range of *M*_*RP_TC*_ is 0 to 100% and higher value implies the better performance.


$$M_{RP\_TC}=\frac{\sum_{j=1}^{m}\left(rf_{i}\;\times \;(\sum_{i=TR_{i}}^{n}tc_{i}\;-\frac{1}{2}*tcTR_{i})\right)}{\sum_{j=1}^{m}rp_{i\;\times \;\;\sum_{j=1}^{n}tc_{j}}}$$ (4)

#### 4.5.5 The average severity of faults detected (ASFD)

The ASFD metric was introduced by Hema Srikanth and Laurie Williams [86]. ASFD value is the ratio of the sum of severities detected by a specific requirement and Total Severity of the Faults Detected (TSFD). It can be represented by Eq 5.


$$ASFD_{i}=\left[\frac{\sum_{j=1}^{m}SV_{j}}{TSFD}\right]$$ (5)

#### 4.5.6 The average percentage of business importance earned (APBIE)

The APBIE was introduced by Qi Li, and Barry Boehm [79]. This metric is proposed to cover the business significance of the system under test. It can be represented by Eq 6.


$$APBIE=\sum_{i=1}^{n}PBIE_{i}/n$$ (6)

### 4.6 Open research problems and recommendations to fill the research gaps (RQ6)

We have conducted this study to highlight open research problems of value-based cost-cognizant TCP techniques and to suggest a few directions on how to improve the reliability of value-based TCP techniques. According to a study [90], cost-cognizant TCP techniques are used when assumptions associated with APFD do not hold. These assumptions are all faults have similar severity and all test cases have similar costs. In practice, these assumptions seldom hold and, in this scenario, the APFD metric does not remain appropriate to evaluate the performance of TCP techniques. Below are a few open research questions related to the evaluation metric selection.

- How often do APFD assumptions hold?
- If the above assumptions rarely hold, then why is the APFD measure highest in popularity in TCP research?
- Is APFDc a good alternative to APFD? If yes, then why APFDc is far behind in comparison with APFD?
- Does the research community need a new standard measure for performance evaluation of TCP techniques?

A limitation of the existing literature is that while using the APFD metric, the researchers did not explicitly mention that basic assumptions of APFD hold or not. They did not mention the reason why they selected APFD as an evaluation metric. Most of the researchers expressed that APFD is the most popular measure which is why we are using it. This is not a strong and valid justification for the metric selection. Therefore, the results produced by using APFD are mostly unreliable in the cases where the assumption “all faults have equal severity and all test cases have equal cost” does not hold [87]. The selection of performance evaluation metrics is still an open research problem. The coverage-based techniques are related to a specific element like statement, condition, branch, methods, or requirements. The metric used for performance evaluation of these techniques is the Average Percentage of Statement Coverage (APSC), the Average Percentage of Condition Coverage (APCC), the Average Percentage of Branch Coverage (APBC), the Average Percentage of Method Coverage (APMC), and Average Percentage of Requirement Coverage (APRC). These metrics can be collectively described as the Average Percentage of Element Coverage (APEC) [67]. These metrics are similar to APFD and are based on the same assumption that all statements, conditions, branches, methods, or requirements are of the same worth, and the test cases used to cover these certain elements have the same cost. This is a major limitation of coverage-based techniques and their utilized metrics, and they might not produce intended results. We suggest that value considerations should be considered in coverage-based techniques and traditional coverage-based metrics should be replaced with value-based coverage metrics. Introducing value in the TCP process can make TCP techniques more efficient and effective.

The existing coverage-based TCP techniques are not aligned with the client’s priorities. The client may bother with some features and may not for others. Some features may involve profitability and productivity for the client business, and some may not. There is a slogan in SE that “Client is always right”. But the client’s perspective is missing in existing TCP techniques. Most of the existing TCP techniques have been proposed in a value-neutral fashion and do not consider the client’s business value expectations. Business value orientation has great potential in the TCP process. Proposed techniques detect a huge number of faults even then releases become late because high severity bugs are detected late in the regression cycle. Debugging and fixing such critical faults at the eleventh hour creates stress on development teams and fixes become prone to further errors. Therefore, detecting critical faults early in the regression testing life cycle is vital. The value-based TCP branch depicted in the taxonomic classification of TCP in Fig 4 is still a gray area. There is great potential for further research related to value orientation in all categories of TCP. Value orientation should be considered in research methods, study contexts, prioritization approaches, and performance evaluation metrics. This is a big surprise that TCP research is continuously coming in a value-neutral fashion despite knowing the fact that all software elements do not have equal worth. Here are a few recommendations to fill the research gap.

A quick paradigm shift from a value-neutral test prioritization to value-based test prioritization is needed. Value-neutral TCP techniques are not likely to produce satisfactory and reliable results. Therefore, TCP remains unable to achieve its intended goals. To fill this major research gap, further research in the domain of TCP should be in a value-based fashion and performance evaluation should be done through value-based cost-cognizant metrics. The study results show that there is a limited application of machine learning techniques in value-based cost-cognizant TCP techniques. Further research should try to solve the TCP problem by applying machine learning techniques to achieve efficiency in the prioritization process. It is also evident that the dataset used for results validation is publicly available only for one study P1. The rest of the studies did not use the public data set for validation. Public datasets should be used so that future researchers can conduct empirical studies to reproduce the results and make further improvements. A limitation is reported in the recent literature that simple statements and traditional coverage cannot guarantee 100% fault detection [67]. Coverage-based techniques are producing less optimistic outcomes. Utilizing value coverage can overcome this limitation. According to the study, most of the work done covers only the functional aspects of the applications [14]. Security, usability, privacy, and performance are very important and perhaps these are not addressed in traditional code coverage metrics. There is a need for coverage of non-functional aspects in the TCP process as well.

## 5. Threats to validity

In this section, we identified a few known threats to the validity of this study’s results. Our defined research questions may not include all aspects of value-based cost-cognitive TCP techniques. This is a construct validity threat and we addressed it through discussions. We believe that our research questions are well designed and mapped with the goals of the study. Ensuring perfection in the data collection process is a difficult task. We cannot guarantee that our data collection is complete. Imperfect data collection can be a threat to the validity of this SLR. We carefully selected our search keywords to fetch more relevant studies from the research repositories. Our paper search was limited to a few prominent research repositories. There might be more relevant publications available in other search repositories. To minimize this problem, we utilized those research repositories which were utilized by previous reviews of TCP techniques. Validation of the study relevancy evaluation process is also a major threat for any SLR. To address this issue, an independent reviewer also evaluated selected studies’ relevance. The second author (supervisor) played this role as an independent reviewer. The data extraction process may be imprecise, and this may affect the validity of this research. It is due to the unsystematic data extraction process. To reduce this risk, we applied manual data extraction through expert judgment. Drafting of a review protocol can also affect the study selection. We followed Barbara Kitchenham’s guidelines to prepare our study protocol to avoid any associated threat.

## 6. Conclusion

The TCP is a vital approach for regression testing to meet time and budget constraints. There are two major classes of TCP techniques 1) Value-neutral TCP techniques 2) Value-based TCP techniques. Both classes have many other categories like coverage-based, history-based, and risk-based. The value-neutral TCP techniques assume that all elements like statements, requirements, test cases, use cases, methods, and bugs are equally important. This assumption rarely holds therefore value-neutral TCP techniques are prone to produce unsatisfactory results. Due to this major limitation of the TCP process, value-based cost-cognizant TCP techniques are gaining popularity. To the best of our knowledge, currently, no review is available on value-based cost-cognizant TCP techniques.

- In this paper, an SLR of value-based cost-cognization TCP techniques is performed. The objective of this study is to see the current state of research in this field.
- This review is evident that there is very limited work on value-based test prioritization. It is needed to realize that without value considerations in the TCP process, its intended results cannot be achieved.
- The right metric selection for the performance evaluation of TCP techniques is essential to get reliable results. Popularity-based metric selection is not a valid justification, and it cannot produce reliable results. This is a big area for further improvement. The efficiency and effectiveness of TCP approaches are strongly dependent on the correct evaluation metric because a researcher usually targets an improvement in a metric value while proposing a TCP technique.

An enhanced taxonomy of TCP techniques has been devised in this SLR for further advancements in the value-based cost-cognizant TCP process. This SLR yields that there is a great potential in value-based cost-cognizant TCP and future research should cover this important dimension.

## Supporting information

S1 Checklist PRISMA 2020 checklist. (DOCX)

S1 Fig PRISMA flow diagram for search process and selection procedure. (DOCX)
