# Supplementary figures and images for: Value-based cost-cognizant test case prioritization for regression testing
Source: PLoS One. 2022 May 17;17(5):e0264972. doi: 10.1371/journal.pone.0264972 (PMC9113597; doi:10.1371/journal.pone.0264972)

**PRISMA FLOW DIAGRAM**


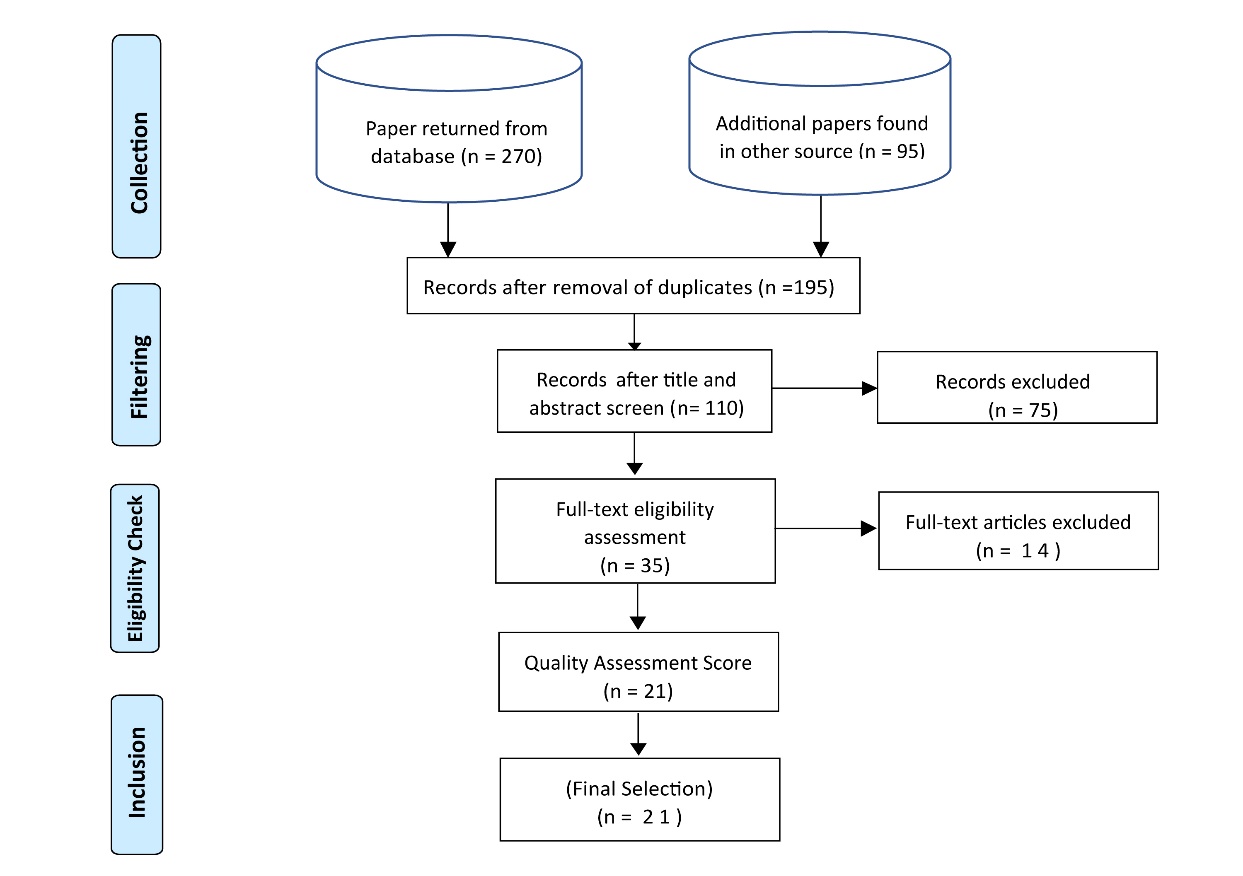


FIGURE 3: PRISMA Flow Diagram for Search Process and Selection Procedure

Supplement: S1 Fig — (DOCX) [file pone.0264972.s002.docx]
